# Formation of a new type of uranium(iv) poly-oxo cluster {U_38_} based on a controlled release of water *via* esterification reaction†
†Electronic supplementary information (ESI) available: Optical photographs, SEM photographs and X-ray diffraction patterns of UO_2_-like powders, UV/visible absorption spectra of supernatant solutions for **2**, thermogravimetric curves, Avrami–Erofeev and Sharp-Hancock kinetic fits, UV-Vis spectra, and time evolution of ^1^H NMR spectra and SAXS curves (PDF). CCDC 1822435 and 1822436 crystallographic data for **1** and **2** (CIF). For ESI and crystallographic data in CIF or other electronic format see DOI: 10.1039/c8sc00752g


**DOI:** 10.1039/c8sc00752g

**Published:** 2018-05-09

**Authors:** Nicolas P. Martin, Christophe Volkringer, Natacha Henry, Xavier Trivelli, Grégory Stoclet, Atsushi Ikeda-Ohno, Thierry Loiseau

**Affiliations:** a Unité de Catalyse et Chimie du Solide (UCCS) , UMR CNRS 8181 , Université de Lille , ENSCL , Bat C7, BP 90108 , 59000 Lille , France . Email: thierry.loiseau@ensc-lille.fr ; Fax: +33 3 20 43 48 95 ; Tel: +33 3 20 434 122; b Institut Universitaire de France (IUF) , 1 rue Descartes , 756231 Paris Cedex 05 , France; c Université de Lille , CNRS , UMR 8576 , UGSF , Unité de Glycobiologie Structurale et Fonctionnelle , F-59000 , France; d Unité Matériaux Et Transformations (UMET) , UMR CNRS 8207 , Université de Lille Nord de France , USTL-ENSCL , Bat C7, BP 90108 , 59652 Villeneuve d'Ascq , France; e Helmholtz-Zentrum Dresden-Rossendorf , Institute of Resource Ecology , Bautzner Landstrasse 400 , 01328 Dresden , Germany

## Abstract

A new strategy for the synthesis of large poly-oxo clusters bearing 38 tetravalent uranium atoms {U_38_} has been developed by controlling the water release from the esterification reaction between a carboxylic acid and an alcohol. The molecular entity [U_38_O_56_Cl_40_(H_2_O)_2_(ipa)_20_]·(ipa)_*x*_ (ipa = isopropanol) was crystallized from the solvothermal reaction of a mixture of UCl_4_ and benzoic acid in isopropanol at temperature ranging from 70 to 130 °C. Its crystal structure reveals the molecular assembly of the UO_2_ fluorite-like inner core {U_14_} with oxo groups bridging the uranium centers. The {U_14_} core is further surrounded by six tetrameric sub-units of {U_4_} to form the {U_38_} cluster. Its surface is decorated by either bridging- and terminal chloride anions or terminal isopropanol molecules. Another synthesis using the same reactant mixture at room temperature resulted in the crystallization of a discrete dinuclear complex [U_2_Cl_4_(bz)_4_(ipa)_4_]·(ipa)_0.5_ (bz = benzoate), in which each uranium center is coordinated by two chlorine atoms, four oxygen atoms from carboxylate groups and two additional oxygen atoms from isopropanol. The slow production of water released from the esterification of isopropanol allows the formation of the giant cluster with oxo bridges linking the uranium atoms at a temperature above 70 °C, whereas no such oxo groups are present in the dinuclear complex formed at room temperature. The kinetics of {U_38_} crystallization as well as the ester formation are analyzed and discussed. SAXS experiments indicate that the {U_38_} species are not dominant in the supernatant, but hexanuclear entities which are closely related to the [U_6_O_8_] type are formed.

## Abstract 2

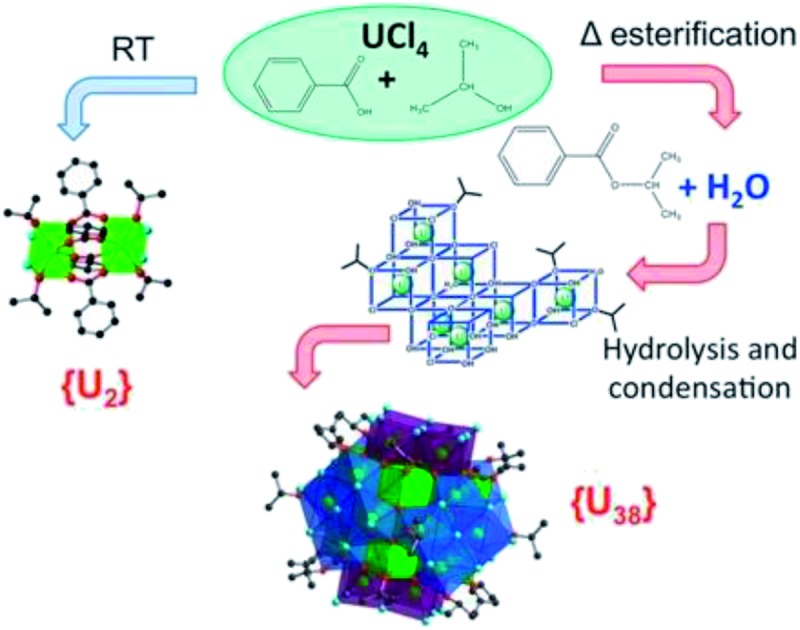
A strategy for the formation of uranium(iv) clusters consists of water release from esterification reaction to generate {U_38_} species.

## Introduction

Since the last decade, many contributions have been devoted to the precise identification of large molecular polynuclear species bearing actinides. These large polynuclear species have been structurally described in the cases of giant cage-like hexavalent uranyl peroxides^1^–^3^ or cluster-like uranium/plutonium oxides^4^–^6^ for their tetravalent oxidation state. Indeed, due to their high ionic charge, tetravalent actinides (An(iv)) exhibit specific properties in a series of actinides with different oxidation states. In addition to their relatively low solubility, they are known to show a strong hydrolysis tendency^7^,^8^ and are easily precipitated through poly-condensation reactions resulting in the formation of amorphous or crystalline solids of AnO_2_·*x*H_2_O type or waterborne colloids.^9^ This particular chemical behavior of An(iv) is of crucial interest from environmental aspects^10^–^12^ since the migration of such radioactive elements is potentially affected by nucleation and crystal growth processes, which may occur in natural groundwater at geological sites (mines or deep waste depositories). Moreover, the stabilization of An(iv) can be favored under anoxic conditions, in contact with reducing biological micro-organisms (bacteria) for instance. With uranium, this could result in the formation of nanocrystalline uraninite (UO_2_) particles with the particle size ranging from 0.9 to 5 nm and large aggregates of a few hundreds of nanometers in size.^13^–^16^

In these contexts, the knowledge of the nature of condensed polyoxo-clusters with different hydrolysis kinetics is required for the understanding of the potential chemical behaviour of such An(iv) clusters in a relevant environment. These clusters can also play an important role as prenucleation species or intermediates for the formation of AnO_2_-type nano-crystallites. Many investigations on these clusters have reported the solid-state crystallization and structural characterization of polynuclear entities, which have been stabilized using ligands (mainly carboxylate) in order to isolate molecular oxo-clusters and prevent any further infinite condensation.^8^ One of the most studied motifs is the hexanuclear coordination complex with the [An_6_O_8_] type, which is known to form with different ligands (sulfate,^17^,^18^ selenate,^19^ phosphonate,^20^ and triflate^21^,^22^). The hexanuclear complex has been described for several An(iv) with different carboxylic acids such as formic acid, acetic acid (Th and U^23^–^25^ in the solid state, and Np in solution^26^), benzoic acid (U),^27^ glycine (Th,^28^,^29^ U,^30^ and Pu^31^) and more recently DOTA (1,4,7,10-tetraazacylododecane-1,4,7,10-tetraacetic acid) ligand with U, Np and Pu.^32^,^33^ Such a hexameric moiety is also encountered as a secondary building unit in the three-dimensional construction of metal–organic frameworks with ditopic carboxylate linkers (Th and U).^34^–^38^ There exist very few motifs with higher nuclearities with Th(iv) ({Th_8_} and {Th_9_}^39^), whereas the exploration in the uranium system revealed the identification of many polynuclear oligomers such as {U_10_},^40^ {U_12_},^22^,^41^ {U_16_}^40^ and {U_38_}.^6^ The latter species have been synthesized by using coordinating ligands, such as benzoate or triflate, in different organic solvents (acetonitrile and tetrahydrofuran) together with the control of water content in the system to monitor the condensation process, leading to the formation of poly-oxo clusters with these different high nuclearities. With Pu(iv), there is much less knowledge regarding the polynuclear bricks, due to its stronger Lewis acid character which results in the rapid formation of precipitates. However, a large anionic moiety of {Pu_38_}^4^,^5^ has been reported, suggesting that a wide range of species with intermediary nuclearities may exist with this element.

In this contribution, we explore a new strategy to produce large polynuclear actinide-oxo moieties of {U_38_} with a nanometer-size by controlling the formation of water released from the esterification reaction between a carboxylic acid and an alcohol. This strategy has been successfully applied to the polycondensation of tetravalent transition metals (Ti and Zr),^42^–^44^ which are highly reactive toward the hydrolysis process. In alcoholic solvents, it was thus possible to prepare a wide variety of well-defined molecular bricks containing oxo/hydroxo groups bridging the metallic centers, Ti^4+^ or/and Zr^4+^.^45^–^47^ Other recent studies have reported the solvothermal synthesis of large polyoxo-titanium cage-like clusters, obtained in the presence of pivalic acid or formic acid in isopropanol solvent, for the production of {Ti_22_}^48^ or {Ti_42_}^49^ motifs, respectively.

We herein extend this synthetic strategy to the An(iv) system to produce new molecular poly-oxo cluster arrangements. In our previous work, we succeeded in the preparation of the large motif {U_38_},^6^ which was obtained from a mixture of uranium tetrachloride (UCl_4_) and benzoic acid in tetrahydrofuran (THF) with the controlled addition of water to induce the oxolation reaction. The resultant product was a crystalline assembly of a distinct moiety containing 38 uranium centers with a fluorite-like arrangement through oxo bridges. The cluster core is further surrounded by chloride and benzoate groups. Our approach in this study is to use the same starting reactants (*i.e.* UCl_4_ and benzoic acid) but in a different organic solvent (isopropanol) without the addition of water. Isopropanol is the best solvent of choice for the successful formation of giant poly-oxo titanium cages, as detailed in this study.

This study deals with the solvothermal synthesis for the formation of a new member of the large poly-oxo cluster {U_38_} by using benzoic acid and isopropanol to achieve the slow *in situ* production of water *via* esterification. The critical role of the esterification process in the formation of the {U_38_} cluster is investigated as functions of different parameters, such as temperature, reaction time and concentration of benzoic acid. It shows that the temperature has a crucial role, since it has a drastic impact on the kinetics of the formation of the cluster {U_38_}. At room temperature, when esterification is not favored, the crystallization of a coordination complex with two uranium atoms, [U_2_Cl_4_(bz)_4_(ipa)_4_]·(ipa)_0.5_ (ipa = isopropanol; bz = benzoate; denoted **1**), is observed. Above 70 °C, the solvothermal treatment gives rise to the formation of the cluster with 38 uranium centers, [U_38_O_56_Cl_40_(H_2_O)_2_(ipa)_20_]·(ipa)_*x*_ (denoted **2**). Their single-crystal structures from XRD analysis as well as synthesis conditions are discussed in the present paper.

## Experimental section

**Caution!** Uranium (U) is a radioactive and chemically toxic element. Special precautions with suitable equipment and facility for radiation protection are required for handling its substances.

### Synthesis

The compounds discussed in this study have been synthesized in a 2 mL glass vial with a Teflon cap by using the following chemical reactants: uranium tetrachloride (UCl_4_, obtained using the protocol based on the reaction of uranium oxide UO_3_ with hexachloropropene^50^), anhydrous isopropanol (C_3_H_8_O, referred to as “ipa” hereafter, purified through a MBraun solvent purification system (MB SPS-800) prior to use), benzoic acid (C_6_H_5_COOH, referred to as “Hbz” hereafter, Aldrich, 99.5%) and de-ionized water. The manipulation of UCl_4_ and the preparation of reactant mixtures were performed in a glove box under an argon atmosphere, while the heating of the reactant mixtures in an oven was carried out under an ambient atmosphere.

#### [U_2_Cl_4_(bz)_4_(ipa)_4_]·(ipa)_0.5_ (**1**)

A mixture of 10 mg of UCl_4_ (0.026 mmol) and 30 mg of benzoic acid (0.24 mmol) in 1 mL of isopropanol (13.1 mmol) was placed in a closed glass vial and then left at room temperature. After 2 weeks, green crystals with a lozenge shape and 150 μm length appeared (Fig. S1a†). The reaction yield is 15% (based on U).

#### [U_38_O_56_Cl_40_(H_2_O)_2_(ipa)_20_]·(ipa)_*x*_ (**2**)

A mixture of 10 mg of UCl_4_ (0.026 mmol) and 30 mg benzoic acid (0.24 mmol) in 1 mL of isopropanol (13.1 mmol) was placed in a closed glass vial and then heated statically at 100 °C for 24 hours. We noticed that the crystallinity of the compound becomes lower when the crystals are removed from the mother liquor and exposed to air, resulting in a color change from orange-brown to black. The resultant crystalline product of **2** (octahedral-shape crystallites – Fig. S1b†) was therefore transferred into its mother liquor in a glove box, and single crystals were picked up for single-crystal X-ray diffraction analysis. The crystals of **2** are very sensitive to air and readily soluble in water, in which they are decomposed. The reaction yield is 37% (based on U).

### Single-crystal X-ray diffraction

Crystals of compounds **1** and **2** were analyzed on a Bruker DUO-APEX2 CCD area-detector diffractometer at 300 K, using microfocus Mo-Kα radiation (*λ* = 0.71073 Å) with an optical fiber as a collimator. The crystals were selected under a polarizing optical microscope and mounted on a MiTeGen™ loop with grease for single-crystal X-ray diffraction experiments. Several sets of narrow data frames were collected (20 s per frame) at different values of *θ* for two initial values of *φ* and *ω* using 0.3° increments of *φ* or *ω*. Data reduction was accomplished using SAINT V7.53a.^51^ The substantial redundancy in data allowed a semi-empirical absorption correction (SADABS V2.10 (ref. 52)) to be applied, on the basis of multiple measurements of equivalent reflections. The structure was solved by direct methods, developed by successive difference Fourier syntheses, and refined by full-matrix least-squares on all *F*^2^ data using the SHELX^53^ program suite with the OLEX2 interface.^54^ Hydrogen atoms of the benzene ring and isopropyl groups were initially placed at calculated positions and allowed to ride on their parent atoms, except those belonging to the disordered organic fragment. The final refinements include anisotropic thermal parameters of all non-hydrogen atoms, except the atoms of some disordered organic groups that have been refined isotropically. The crystal data are given in Table 1. The crystal structures are available in CIF format (CCDC file numbers: ; 1822435 and ; 1822436).†

**Table 1:** Crystal data and structure refinements for [U_2_Cl_4_(bz)_4_(ipa)_4_]·(ipa)_0.5_ (**1**) and the poly-oxo uranium cluster {U_38_} (**2**)

	**1**	**2**
Formula	C_41.5_H_56_Cl_4_O_12.5_U_2_	C_60_H_148_Cl_42_O_76_U_38_
Formula weight	1372.72	12 619.81
Temperature/K	300	300
Crystal type	Green plate	Brown octahedron
Crystal size/mm	0.10 × 0.09 × 0.08	0.133 × 0.11 × 0.083
Crystal system	Triclinic	Tetragonal
Space group	*P*1[combining macron]	*I*4/*m*
*a*/Å	10.683(2)	19.6962(7)
*b*/Å	11.322(2)	19.6962(7)
*c*/Å	11.736(2)	29.9535(13)
*α*/°	67.493(9)	90
*β*/°	86.541(10)	90
*γ*°	76.417(10)	90
Volume/Å^3^	1273.9(4)	11 620.2(1)
*Z*, *ρ*_calculated_/g cm^–3^	1, 1.789	2, 3.607
*μ*/mm^–1^	6.612	26.904
*Θ* range/°	1.879–27.103	2.287–28.070
Limiting indices	–13 ≤ *h* ≤ 13	–22 ≤ *h* ≤ 26
	–14 ≤ *k* ≤ 14	–24 ≤ *k* ≤ 23
	–15 ≤ *l* ≤ 15	–38 ≤ *l* ≤ 39
Collected reflections	38 899	47 568
Unique reflections	5575	7301
	[*R*(int) = 0.0407]	[*R*(int) = 0.0657]
Parameters	348	266
Goodness-of-fit on *F*^2^	1.050	1.017
Final *R* indices [*I* > 2*σ*(*I*)]	*R*_1_ = 0.0246	*R*_1_ = 0.0409
	w*R*_2_ = 0.0595	w*R*_2_ = 0.0749
*R* indices (all data)	*R*_1_ = 0.0303	*R*_1_ = 0.1188
	w*R*_2_ = 0.0615	w*R*_2_ = 0.0942
Largest diff. peak and hole/e Å^–3^	1.68 and –0.73	3.030 and –1.576

### Thermogravimetric analysis

The thermogravimetric experiments have been carried out on a thermo-analyzer TGA 92 SETARAM under an argon atmosphere with a heating rate of 5 °C min^–1^ from room temperature up to 800 °C.

### UV-visible absorption spectroscopy

UV-visible absorption spectra of the supernatant solutions for the synthesis of compound **2** have been collected by using a Perkin-Elmer Lambda 650 spectrophotometer.

### Solution ^1^H nuclear magnetic resonance (NMR) spectroscopy

The supernatants obtained after the crystallization of {U_38_} have been analyzed by solution ^1^H-NMR at 285 K and 9.4 T, on a Bruker Avance II 400 spectrometer equipped with a z-gradient TBI 5 mm probe. The solution samples have been introduced into coaxial inserts (Eurisotop, Saint-Aubin, France) placed in standard 5 mm tubes containing deuterated acetonitrile (CD_3_CN, isotopic enrichment 99.8%, Eurisotop, Saint-Aubin, France) used for the lock and shim settings. Chemical shifts in parts per million (ppm) are referenced to the residual solvent peak of CHD_2_CN at 1.94 ppm. An additional peak is also observed at 2.35 ppm and assigned to residual water in deuterated acetonitrile.

### Small-angle X-ray scattering (SAXS) measurements

The measurements were performed on a SAXS Xeuss 2.0 apparatus (Xenocs) equipped with a micro X-ray source with Cu Kα radiation (*λ* = 1.5406 Å) and point collimation (beam size: 300 × 300 μm^2^). The sample-to-detector distance (approximately 35 cm) was calibrated using silver behenate as a standard. The sample solutions were placed in 1.5 mm glass capillaries at a concentration of approximately 25 mM U. Prior to each measurement, the background contribution of the capillaries and the solvent was subtracted using a reference containing isopropanol according to standard procedures.

## Results

### Structure description

**Fig. 1:**
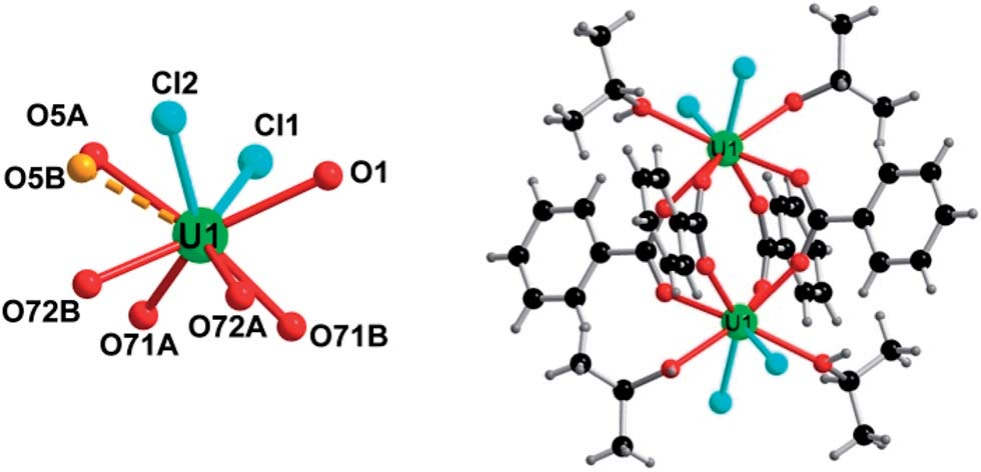
Representation of the coordination surrounding of the uranium atom (left) and the dinuclear entity (right) in the complex [U_2_Cl_4_(bz)_4_(ipa)_4_]·(ipa)_0.5_ (**1**). (left) The oxygen atoms O5A and O5B belong to disordered isopropanol ligands. (right) For clarity, the disorder of the isopropanol molecule is not shown. Color code: green: uranium, red: oxygen, cyan: chlorine, dark grey: carbon, and light grey: hydrogen.

**Fig. 2:**
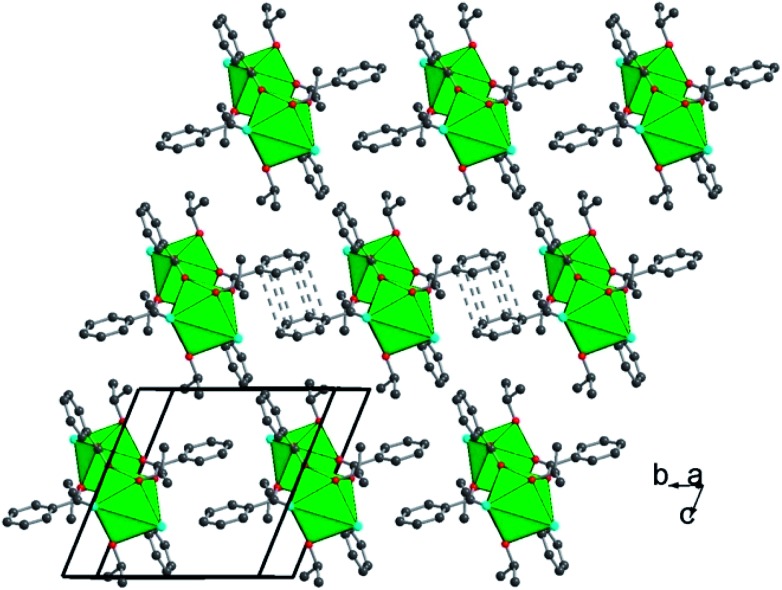
Polyhedral view of the structure of [U_2_Cl_4_(bz)_4_(ipa)_4_]·(ipa)_0.5_ (**1**) along the (*b*, *c*) plane, showing the stacking of the neutral dinuclear entities. Dotted lines between carbon atoms of the benzene rings of the monocarboxylate molecule indicate the π–π interactions. For clarity, the disorderly attached isopropanol ligand and intercalated isopropanol molecules are not shown. Color code: green polyhedra: uranium, red: oxygen, cyan: chlorine, and dark grey: carbon.

Two distinct compounds have been isolated from the mixture of UCl_4_ and benzoic acid in isopropanol. A first coordination complex (**1**), obtained at room temperature, reveals a molecular structure containing two uranium centers linked by four carboxylate bridges of the benzoate molecules (Fig. 1). In this dinuclear unit, the unique crystallographically independent uranium atom is eight-fold coordinated in a square antiprismatic geometry, with four carboxyl oxygen atoms (U–O = 2.299(3)–2.340(3) Å), two oxygen atoms from the isopropanol molecule (U–OH = 2.475(13)–2.515(5) Å) and two terminal chlorine atoms (U–Cl = 2.6698(12)–2.7183(10) Å). The uranium centers are arranged around a symmetry center (–1), resulting in the formation of the dimeric species. The isopropanol and chlorine molecules are located at the periphery of the dinuclear unit, whereas the benzoate molecules are situated in a central position and bridge the two uranium atoms with a *syn*–*syn* bidentate connection mode. One of the two attached isopropanol ligands is found to be disordered on two close positions, with an occupancy factor of 75/20 (Fig. S3a†). The bond valence sum (BVS) calculations^55^ indicate the uranium atom in the tetravalent state (3.991). Complex **1** with a formula [U_2_Cl_4_(bz)_4_(ipa)_4_]·(ipa)_0.5_ is neutral, in which four chloride and four benzoate anions compensate the positive charges of the two tetravalent uranium atoms. Indeed, no direct bonding *via* oxo or hydroxo groups is observed between the uranium atoms, and only the carboxylate arms of the benzoate groups act as linkers for these species. The crystal structure is formed through the stacking of the dimeric motifs, which occurs through weak π–π interactions (C···C ≈ 3.8 Å) between the benzene rings of the monocarboxylate molecules (Fig. 2). A free isopropanol is intercalated in the dinuclear complex [U_2_Cl_4_(bz)_4_(ipa)_4_] and observed to be disordered on two close positions (occupancy factor set at 0.25; Fig. S3b†). It interacts *via* hydrogen bonds between its OH group and the OH group of the isopropanol linked to uranium (O–H···O–H ≈ 2.54 Å).

**Fig. 3:**
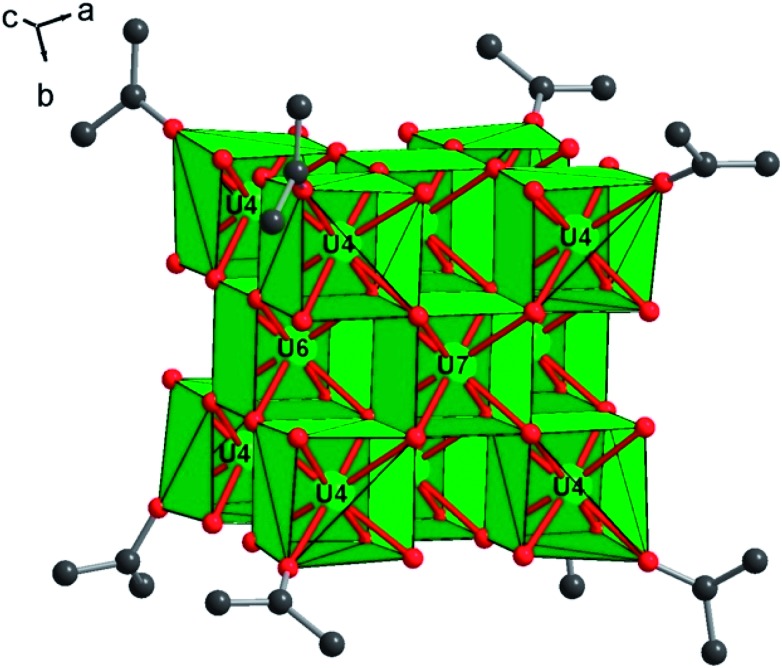
View of the internal {U_14_} core in compound **2**, showing the cube-like arrangement of the uranium-centered UO_8_ polyhedra (U4, U6, and U7). Each edge of the {U_14_} motif is occupied by an isopropanol molecule, whose carbon atoms are statistically located at two disordered positions. For clarity, only one position of isopropanol is shown in the figure. Color code: green polyhedra: uranium, red: oxygen, and dark grey: carbon.

Heating the reactant mixture resulted in the formation of a second compound (**2**). It crystallizes in the tetragonal symmetry (*I*4/*m*). The analysis of its structure is rather complex since it consists of the molecular assembly of poly-oxo clusters containing 38 uranium centers. In this sense, the atomic arrangement is reminiscent of that of the {U_38_} cluster^6^ synthesized in tetrahydrofuran (THF) with an identical crystal symmetry. However, due to the absence of benzoate linkers, fewer polyhedral distortions are observed in **2**. The new {U_38_} cluster (**2**) is based on the dense association of uranium atoms in a fluorite-like arrangement, which contains 14 uranium cations (three crystallographically independent atoms, U4, U6 and U7) surrounded by eight oxygen atoms in a cubic polyhedral coordination of UO_8_ (Fig. 3). Within the internal {U_14_} core, the U–O bond lengths range from 2.328(6) to 2.376(5) Å for U6 and U7, and all the oxygen atoms adopt a μ_4_-coordination mode. The polyhedron around the uranium U4 located at each corner of the {U_14_} pseudo-cube is slightly distorted with internal U–O bond distances varying between 2.312(6) and 2.424(6) Å, a longer U–O distance with the oxygen belonging to a terminal isopropanol molecule (U–O_ipa_ = 2.445(8) Å). The carbon atoms of the isopropanol are statistically disordered on two equivalent positions, pointing outward the {U_14_} pseudo-cubic motif along the [111] direction. In the former {U_38_} cluster synthesized in THF, the external oxygen atom belonged to the THF molecules, which are replaced by isopropanol in the present species.

**Fig. 4:**
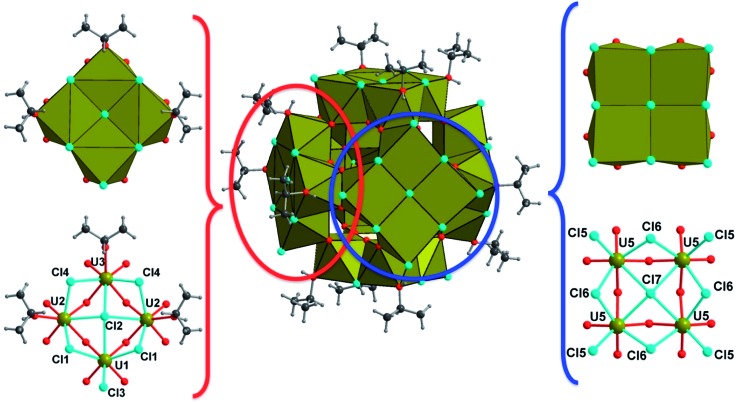
Detailed view of the tetranuclear sub-units decorating the periphery of the {U_38_} cluster in compound **2**. The internal {U_14_} fluorite core is not shown in the figure for clarity. The representation shows the two types of tetranuclear sub-units (U1, 2 × U2, and U3 (left), and 4 × U5 (right)), with either terminal isopropanol molecules (left) or terminal chlorine atoms (right). Color code: khaki polyhedra: uranium, red: oxygen, cyan: chlorine, dark grey: carbon, and light grey: hydrogen.

The {U_14_} moiety is surrounded by six sets of tetranuclear {U_4_} sub-units, which are located at each of its faces (Fig. 4). Two types of {U_4_} entities are observed, following the tetragonal 4 axis symmetry (for U5) or mirror *m* symmetry (for U1, U2 and U3). The U5-containing tetramer is repeated twice through mirror symmetry and consists of uranium centers in an eight-fold coordination. The uranium atom U5 is observed in a distorted square anti-prismatic geometry, with four U5–O bonds (2.231(6)–2.236(6) Å for μ_3_-oxo, and 2.362(6)–2.371(6) for μ_4_-oxo) from oxygen atoms bridging U4 and U7 centers of the internal {U_14_} core and U2 centers from adjacent {U_4_} sub-units. They are four contrasting U–Cl bonds (2.712(3)–3.084(2) Å), resulting in the formation of a UO_4_Cl_4_ species. We notice the longer U–Cl distances of 3.084(2) Å with a μ_4_ coordination bridging (Cl7), whereas the other chlorine atoms adopt either a bridging μ_2_ coordination mode (U5–Cl6 = 2.809(3)–2.831(3) Å) or a terminal mode (U5–Cl5 = 2.712(3) Å). The second type of tetrameric sub-unit, repeated four times, is based on the connection of two U2 atoms with one U1 atom and one U3 atom through oxo- and chloro-bridges. The three crystallographically independent uranium atoms are eight fold-coordinated in the anti-prismatic polyhedral geometry. U1 (as observed for U5) is linked to four oxygen atoms (U1–O = 2.202(6) Å for μ_3_-oxo and 2.360(6) Å for μ_4_-oxo) and four chlorine atoms, which are either in μ_4_-bridging (U1–Cl2 = 3.065(4) Å), or in μ_2_-bridging (U1–Cl1 = 2.850(3) Å) or in a terminal fashion (U1–Cl3 = 2.699(4) Å). The three other cations are bound to four oxygen atoms (U–O = 2.199(6)–2.217(6) Å for μ_3_-oxo and 2.315(6)–2.345(6) Å for μ_4_-oxo), three chlorine atoms (U–Cl(μ_2_) = 2.844(3)–2.855(3) Å; U–Cl(μ_4_) = 3.057(4)–3.0615(15) Å) and one oxygen from the isopropanol molecule which exhibits a longer U–O bond length (U–O_ipa_ = 2.409(9)–2.430(7) Å). Within the inner fluorite fragment {U_14_}, the uranium–uranium distances are in the range between 3.848(1) and 3.931(1) Å, and they are closely related to those existing in the dense UO_2_ structure type (U···U = 3.866 Å).^56^

**Fig. 5:**
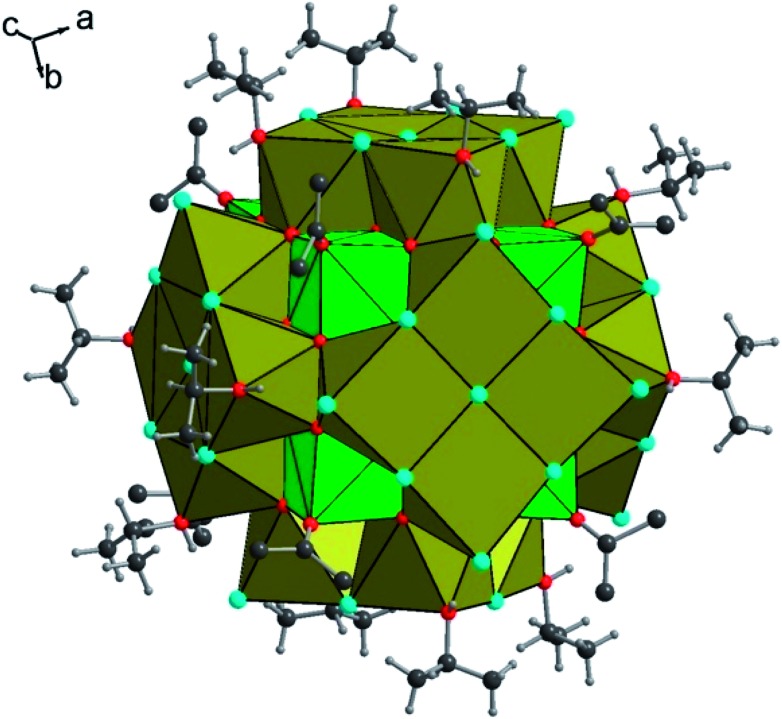
Polyhedral representation of the poly-oxo cluster {U_38_} in compound **2**. Polyhedra indicate the uranium-centered units, which are distinguished as green cubes (internal {U_14_}), and khaki square antiprisms (peripheral {U_4_}) for clarity. Color code: red: oxygen, cyan: chlorine, dark grey: carbon, and light grey: hydrogen.

In total, the cluster contains 38 uranium centers ({U_14_} + 6 × {U_4_}) connected to each other through 56 inner oxo and 42 peripheral chloro groups (Fig. 5). There are 20 isopropanol molecules, in which 8 are attached to the {U_14_} and the rest 12 are attached to four {U_4_} sub-units. The bond valence sum (BVS)^55^ has been calculated for the seven crystallographically independent uranium centers, suggesting the charge of uranium centers varying from 3.905 up to 4.168, which is in good agreement with the tetravalent oxidation state (Table S1†). Based on this consideration, the total charge of the cluster is expected to possess two negative charges with the chemical formula [U_38_O_56_Cl_42_(ipa)_20_]^2–^ deduced from the crystal structure analysis. The same statement was reported for the former {U_38_} cluster obtained from THF.^6^ The hypothesis of the substitution of two chlorine atoms located at the surface of the {U_38_} moiety by two water molecules could be considered, due to the fact that the {An_38_} clusters are reported with different molar amounts of Cl/H_2_O, especially for the {Pu_38_} analogue^4^,^5^ that occurs with two distinct Cl/H_2_O ratios depending on the number of counter-cationic species present in the crystal structure. In this case, a disorder with the ratio 40Cl/2H_2_O could occur, but cannot be visualized accurately by X-ray diffraction due to the high symmetry observed in this compound. Based on this hypothesis, the resulting chemical formula of the new {U_38_} cluster is [U_38_O_56_(H_2_O)_2_Cl_40_(ipa)_20_], since no additional cationic species are present in our reaction medium or they are not visible in the XRD analysis. In another hypothesis, hydroxyl groups may occur in the μ_3_-oxygen atoms located at the periphery of the cluster {U_38_} and bridging the U4 centers (from the {U_14_} core to the U1, U2, U3 and U5 centers from the {U_4_} sub-units). In this case, 24 oxygen atoms are considered, in which 2 could be assigned to hydroxyl groups. This results in a neutral molecular cluster. This disordering O/OH situation was encountered in the hexanuclear brick, for which half of the μ_3_-oxygen atoms are either O or OH groups. Two cases are reported in the literature: either an average U–O bonding (≈2.32 Å (ref. 35)) is observed due to the high symmetry of the crystal structure (ex: cubic), or a disorder of O/OH is clearly visible with two sets of U–O bonds related to longer U–O distances (2.40–2.50 Å (ref. 23, 27, 30 and 34)) for μ_3_-OH and shorter U–O distances (2.20–2.27 Å (ref. 21–23, 27, 30 and 34)) for μ_3_-O. In the latter case, the oxygen atoms are delocalized on two close positions with 50% occupancy. In the new {U_38_} cluster, due to the low occupancy of OH, it was not possible to accurately assign the oxygen sites to the two hydroxyl groups, which could partially occupy the different μ_3_-oxo atoms at the periphery. In this configuration, the chemical formula of the {U_38_} cluster would be [U_38_O_54_(OH)_2_Cl_42_(ipa)_20_]. Without any clear evidence between the two situations discussed above, we arbitrarily chose the first hypothesis with the partial occupancy of water molecules on the terminal chloro groups for the chemical formula of the {U_38_} cluster in the present contribution. Moreover, some similarities exist between compound **2** and the {Pu_38_} cluster;^4^,^5^ the chemical formula differs only by the number of isopropanol molecules, which are replaced by water in the {Pu_38_} cluster. The presence of isopropanol molecules on only four faces of the {U_38_} cluster breaks the symmetry down to the tetragonal one (*I*4/*m*), instead of the cubic one (*Ia*3[combining macron]) observed in the Pu-analogue. In the {Pu_38_} case, some lithium cations were intercalated between the Pu motifs for charge compensation of the crystal structure. In compound **2**, the stacking of the clusters {U_38_} within the crystal cell reveals the presence of some voids (2216 Å^3^), which may reflect the encapsulation of other molecules such as isopropanol solvent or benzoic acid molecules. This is a rather difficult issue to further discuss, since no electronic density residue related to the positions of such trapped molecules is clearly observed from the Fourier map examination. The estimated size of the {U_38_} cluster is in the range of 1.6–1.7 and 2.24 nm, considering the peripheral chlorine–chlorine distances and isopropanol–isopropanol distances, respectively.

There is a possibility of the encapsulation of free isopropanol solvent species in compound **2**, as it was also observed in the {U_38_} cluster obtained in THF.^6^ Therefore, the molecular content of compound **2** was further estimated by elemental- and thermogravimetric analyses. The elemental analysis estimates the amounts of carbon to be 8.75% and hydrogen to be 1.61%, which indicate the presence of ≈13 free isopropanol species per U_38_ unit (C: 8.85% and H: 1.72%). The thermogravimetric curve (Fig. S4†) of compound **2** under an ambient atmosphere indicates a three-step event for the weight loss. The first step occurs between room temperature and 90 °C with a weight loss of 3.4%. This can be assigned to the release of free isopropanol molecules corresponding to 7 species per U_38_ unit. The second weight loss is observed between 90 and 350 °C (6.2%), followed by the third weight loss up to 430 °C (7.8%). At higher temperature, a plateau is clearly visible and related to the formation of the uranium oxide. The powder X-ray diffraction pattern analysis indicates the formation of U_3_O_8_ (PDF no. 00-047-1493). The remaining weight value is 81.9% and agrees well with that calculated for U_3_O_8_ (81.7%), when the chemical formula [U_38_O_56_(H_2_O)_2_Cl_40_(ipa)_20_]·7ipa is considered. The difference between these two analyzes (13 ipa from elemental analysis and 7 ipa from TG) might originate from the technical difficulty to manipulate the crystals of compound **2** for the measurements, since they are unstable in air and very sensitive to moisture. A part of the free solvent molecules can be released with the loss of crystallinity, making it difficult to get the correct molar content of the encapsulated species between the {U_38_} clusters.

### Formation of the poly-oxo cluster {U_38_} in isopropanol

#### Kinetic study of the crystallization of the {U_38_} cluster in isopropanol

**Fig. 6:**
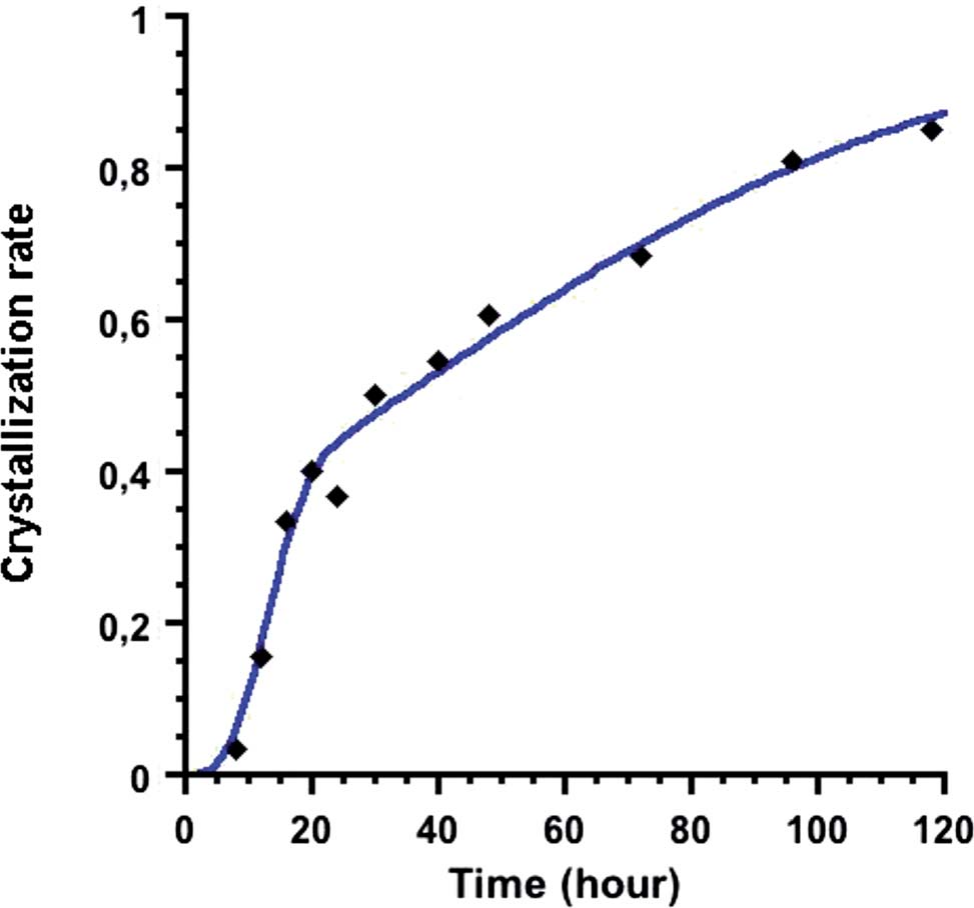
Time variation of the crystallization rate of the {U_38_} polyoxo cluster (heating at 100 °C) between 8 and 120 hours. Black lozenges: experimental data; blue curve: calculations using the Gualtieri equation.

The kinetics of the formation of {U_38_} crystals has been further investigated based on the yield of the products obtained from the mixture of UCl_4_ and benzoic acid in isopropanol heated at 100 °C. The reaction yield was calculated by weighing the mass of the produced {U_38_} crystals as a function of time from *t* = 8 up to 120 h (5 days) after separating the solid products from the supernatant in a glove box. A large amount of the supernatant solution was removed by using a Pasteur pipet, and the {U_38_} crystals were washed with anhydrous isopropanol and dried under reduced pressure in the antechamber of the glove box prior to weighing. Due to the instability of the crystals and technical difficulty to estimate the exact content of isopropanol, the yield calculation was performed based on the arbitrarily estimated chemical formula of {U_38_}, [U_38_O_56_(H_2_O)_2_Cl_40_(ipa)_20_]·7ipa, obtained from the thermogravimetric analysis. Fig. 6 indicates the kinetic curve of the crystal formation of {U_38_} (crystallization rate *α*, scaled from 0 at the beginning and 1 at the end, being related to 0 and 100% yields, respectively). After the initial reaction induction time, the first visible formation of the {U_38_} crystals was observed at *t* = 8 h (reaction yield = 3.3%). The amount of the {U_38_} crystals was constantly increasing afterward, with a reaction yield of 85.0% after 120 h (5 days) of the solvothermal treatment. In a first approximation, the kinetic curve was simulated by assuming the crystallization rate *α* based on the Avrami–Erofeev equation (*α*(*t*) = 1 – exp[–*k*(*t* – *t*_0_)^*n*^]) with a least-squares refinement. This is a kinetic expression that has been originally used in solid-state chemistry for the phase transition or crystal growth, but has been also employed for the interpretation of the crystallization of zeolites,^57^,^58^ metalphosphates^59^–^61^ or coordination polymers^62^–^64^ from homogenous solution under hydro/solvo-thermal conditions. The Avrami–Erofeev equation defines three variable parameters of the induction time (*t*_0_), the rate constant (*k*) and the Avrami coefficient (*n*) to describe an exponential expression of the crystallization rate (*α*). For the crystallization process of the {U_38_} cluster at 100 °C (Fig. S6a†), the overall rate constant is calculated to be *k* = 0.09(2) h^–1^, the Avrami coefficient is *n* = 0.64(5), and the induction time *t*_0_ is 7 h and 48 min with a correlation coefficient *R*^2^ of 0.984 (the fitting was performed using Kaleidagraph 4.5). The validity of this fit can be confirmed by applying a linearization method of the Avrami–Erofe'ev equation, developed by Sharp and Hancock.^65^ Such a plot can be readily obtained using the equation: ln[–ln(1 – *α*)] = *n* ln(*k*) + *n* ln(*t* – *t*_0_), which allows the extraction of the Avrami coefficient *n* and the overall rate constant *k* from the slope and the intercept values of the obtained straight line, respectively. The plot obtained for the {U_38_} formation (Fig. S6b†) shows a linear relationship between ln[–ln(1 – *α*)] and ln(*t* – *t*_0_) with a correlation coefficient *R*^2^ of 0.982, calculating the *k* and *n* values of 0.022(2) h^–1^ and 0.64(3), respectively. Although the rate constant differs between these two fitting analyses, we obtained the same Avrami coefficient *n* of 0.64. This parameter can therefore be used for the interpretation of the reaction mechanism in the crystallization process (*e.g.* diffusion-control, phase-boundary-control, and nucleation). According to the literature,^59^,^66^ the *n* value ranging between 0.5 and 1 indicates that the crystallization mechanism is controlled by the diffusion of reactive species to the crystal. However, another kinetic model introduced by Gualtieri^67^ can also be used to distinguish between the nucleation and the growth processes during crystallization in solution, which is not considered in the Avrami–Erofeev equation. Gualtieri's model has been recently applied to the crystalline formation of porous coordination polymers,^68^–^70^ and expressed by the equation: *α*(*t*) = [1 – exp(–(*k*_G_*t*)^*m*^)]/[1 + exp(–(*t* – *a*)/*b*)], where *k*_G_ is related to the rate constant of the crystal growth (different from *k* in Avrami's model), *a* and *b* are constant values related to the nucleation process, and *m* is related to the dimensionality of the crystal growth. Considering the octahedral shape of the {U_38_} crystals showing a high symmetry, the *m* value was set at 3 in our calculation. The rate constant of nucleation *k*_N_ can then be deduced from the value of *a* as *k*_N_ = 1/*a*. From our experimental kinetic curve shown in Fig. 6, we extracted the different variables of *a* = 34(3) hours, *b* = 44(5) hours, *k*_G_ = 0.071(5) hours^–1^ and *k*_N_ = 0.029(3) hours^–1^ with a correlation coefficient of *R*^2^ = 0.986. The obtained *k*_N_ value is lower than the *k*_G_ one, indicating that the nucleation is the limiting step for the crystallization of the {U_38_} cluster. The parameter *b* higher than 20 indicates that the nucleation follows an autocatalytic process in the present case, based on Gualtieri's theory.^67^

The kinetics of the crystallization of {U_38_} is strongly dependent on the reaction temperature. For instance, the formation of the {U_38_} cluster was visible only after 1 h 30 min when treated at 130 °C, while the formation required 3–4 and 7 days at 80 and 70 °C, respectively. The crystallization of {U_38_} is therefore thermally favored, since the formation of the dinuclear complex [U_2_Cl_4_(bz)_4_(ipa)_4_]·(ipa)_0.5_ (**1**) is dominant at room temperature.

#### Role of the carboxylic acid in the formation of the {U_38_} cluster

As revealed by the XRD analysis, the structural entity of {U_38_} contains uranium centers linked through μ_3_- and μ_4_-oxo groups. However, the cluster was obtained from the mixture of UCl_4_ and benzoic acid in isopropanol without an addition of water, the presence of which generally induces the oxolation/olation reactions required to form oxo bridges *via* hydrolysis. Moreover, the benzoate ligands, which were observed in the {U_38_} cluster obtained in THF,^6^ are not observed in the crystal structure of the present {U_38_} cluster. In fact, in the present case, the oxo groups are actually generated *in situ* by the esterification reaction between the carboxylic acid (benzoic acid) and alcohol (isopropanol). Such an esterification reaction results in the formation of isopropyl benzoate ester together with the release of one water molecule per mole of ester, as shown in Fig. S2.†

**Fig. 7:**
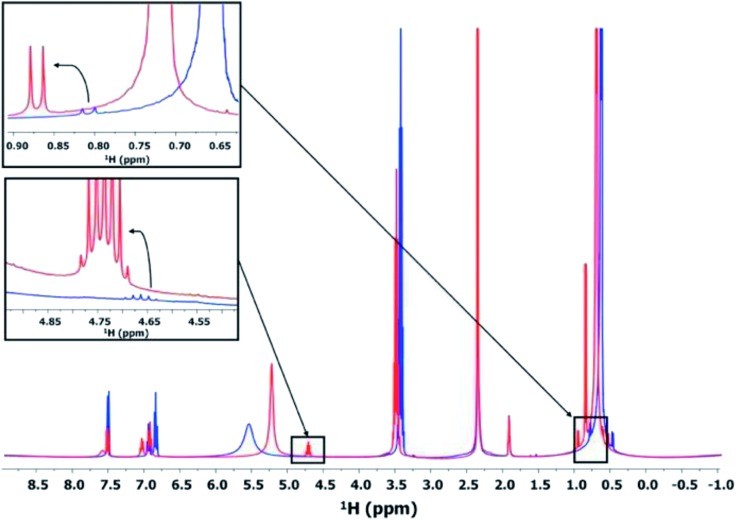
^1^H NMR spectra of the supernatants obtained after the solvothermal reaction (100 °C for 24 h) of the mixture of UCl_4_ and benzoic acid in isopropanol (red) and benzoic acid in isopropanol (blue). The data were recorded at 400 MHz and 285 K. Inset: zoomed-in observation of the –CH and –CH_3_ signal range for highlighting the ester formation.

This synthesis route involving esterification allows a slow and controlled release of water during the treatment, eventually realizing the slow and controlled hydrolysis of U(iv) to form the poly-oxo {U_38_} cluster. The formation of such an ester was confirmed by measuring the liquid ^1^H NMR of the supernatants obtained after the treatment at 100 °C. The ^1^H NMR spectrum of the supernatant after 24 h of the treatment (using the same protocol described in the synthesis section) is shown in Fig. 7. The spectrum shows the peaks corresponding to the –CH group of the benzene ring (6.75–7.75 ppm) and a less intense broad signal attributed to the formation of an uranium benzoate (7.6 ppm). The isopropanol molecule shows its –OH group at around 5–5.75 ppm, the –CH group at around 3.5 ppm, and –CH_3_ groups in the range between 0.25 and 1 ppm. The formation of the ester can be confirmed by the appearance of new signals corresponding to its –CH and –CH_3_ groups. Indeed, a doublet appears at 0.8–0.9 ppm for the –CH_3_ group of the ester, as well as a sextuplet at around 4.65–4.85 ppm for the –CH group. Interestingly, the formation of the ester is favored when UCl_4_ is present in the initial reaction medium. In fact, the peaks corresponding to the –CH_3_ and –CH resonances become weaker in the absence of UCl_4_, indicating less formation of the ester. This phenomenon can be explained by assuming the consumption of water released from the esterification process to form oxo bridging between uranium atoms, which eventually shifts the reaction equilibrium toward the formation of the isopropyl benzoate ester. The Lewis acid character of UCl_4_ may also play a concomitant role as a catalyst in the esterification reaction.

As shown in Fig. S7,† the amount of the formed ester molecules increases with time. When focusing on the range between 4.75 and 5.5 ppm of the obtained ^1^H NMR spectra, we observed a shift of the –OH signal of isopropanol from 5.2 to 5.12 ppm at *t* = 8 and 24 h, respectively. The sextuplet assigned to the –CH group of the ester appeared firstly on the shoulder of the –OH peak at around 5.07 ppm, and the intensity of the sextuplet was increased with increasing the reaction duration and shifted to around 4.92 ppm. These results indicate that the amount of ester increased with increasing the reaction time, which is also related to the increase in the reaction yield for the formation of the poly-oxo {U_38_} cluster. The crystallization of the {U_38_} cluster is therefore directly controlled by the quantitative release of reactive water from the esterification.

In the present study, the initial molar ratio of benzoic acid/uranium (Hbz/U) in the reaction mixture was 9.23 (0.24 mol/0.026 mol). Assuming that the esterification reaction is complete and the esterification of one benzoic acid molecule produces one water molecule, the total amount of water released from the esterification should be equal to the initial amount of benzoic acid. Hence, the final molar ratio of water/uranium (H_2_O/U) is expected to be 9.23. The O/U ratio in the crystal structure of the {U_38_} cluster is 58/38 (=1.53 oxo per uranium atom) and an excess of water should remain in the reaction medium. We therefore analyzed the influence of the initial amount of benzoic acid in the reaction medium on the reaction yield (Fig. S8†). The reaction mixture was treated at 100 °C for 24 h, and the reaction yield was monitored as a function of the initial amount of benzoic acid with the Hbz/U ratio ranging from 1 to 100. The reaction yield was found to be 13% for Hbz/U = 1, which was further increased to 37% for Hbz/U = 10. Surprisingly, the reaction yield decreased drastically to 25% for Hbz/U = 20, and then continuously decreased down to 9% for Hbz/U = 100. The first increasing trend is attributed to the enhanced formation of the {U_38_} crystals by the increase of water molecules released from esterification. The second decreasing trend may originate from two possible chemical processes preventing the formation of the {U_38_} clusters. The first process is related to the preferred formation of uranium benzoate complexes in the reaction mixture with increasing the benzoic acid concentration, as observed at room temperature. In this case, a part of uranium in the initial reaction mixture interacts with benzoic acid to form uranium benzoate complexes including dinuclear complexes (*e.g.***1**). These benzoate complexes are expected to be stable and, therefore, there would be a competition between the formation of the benzoate complexes and the poly-oxo polymers/clusters. The second process is the dissolution of the {U_38_} cluster, which is induced by the increase of water in the reaction system. Indeed, we confirmed that the crystals of {U_38_} are rapidly dissolved when in contact with a drop of water. The hydrolytic stability of the present {U_38_} cluster species is rather weak as compared with that of the {U_38_} cluster obtained in THF.^6^ In this process, the crystallization of {U_38_} would be limited by the solubility limit in the water/isopropanol mixture.

The results discussed above show a significant role of benzoic acid in controlling the formation of the {U_38_} clusters by a slow and controlled release of water *via* esterification. In the present {U_38_} cluster, benzoate molecules are not observed in the final crystal structure, while they are found in the crystal structure of the former {U_38_} cluster synthesized in THF. Additional attempts were also carried out with other types of carboxylic acids. For instance, under the same synthetic conditions of heating at 100 °C for 24 h, the use of formic acid (10 μL, 0.26 mmol) or methacrylic acid (20 μL, 0.23 mmol) instead of benzoic acid also led to the formation of brownish octahedral shaped crystals (Fig. S1c†), which are typical of the {U_38_} crystals. The other attempts to remove the source of the carboxylic acid in the reaction medium and to add water from 5 up to 50 μL (H_2_O/U ratio of 10 or 100) also resulted in the formation of some brown octahedral shaped crystallites assigned to the {U_38_} cluster, with an additional formation of fine dark brown gel-like powder at the surface of the glass vial. An optical microscopy examination still shows the formation of some brown octahedral shaped crystallites, assigned to the cluster {U_38_}, but the major part is constituted by a fine dark brown gel-like powder, observed at the surface of the glass tube wall. The SEM analysis of this fine powder indicates the formation of spherical particles of 0.5–2 μm diameter (Fig. S9a†). Its X-ray diffraction pattern is typical of the dense fluorite-type signature, even if the Bragg peaks are quite broad and of low intensity (Fig. S9b†), due to the presence of partly crystallized and submicron sized particles. This finely powdered product is attributed to a hydrated uranium(iv) dioxide UO_2_·*x*H_2_O and its preparation has been previously reported in the literature through a synthetic route using organic surfactant molecules for instance.^71^ This UO_2_·*x*H_2_O form is known to be formed kinetically as a result of the hydration of U(iv), which is expected at the early stage of the reaction. Such a dense uranium dioxide product was also observed during the formation of the {U_38_} cluster in THF when a controlled amount of water was added.^41^ In this case, the {U_38_–THF} complex was produced after the slow dissolution of the dense product, which further reacted with the carboxylic acid. In the isopropanol system, the additional UO_2_·*x*H_2_O phase is in equilibrium with the {U_38_} phase and is not formed when a slow release of water from the esterification process occurs. This indicates that the formation kinetics of the poly-oxo {U_38_} cluster is rather slower than that of the dense uranium dioxide UO_2_·*x*H_2_O phase. Indeed, another kinetic study suggested that the formation of {U_38_} is limited by the nucleation step rather than by the growth process, and would explain the competition between the formation of UO_2_·*x*H_2_O and {U_38_} compounds. These results also indicate that the nature of the carboxylic acid plays no critical role in the production of the {U_38_} cluster. We also leave the same reactional medium containing water (H_2_O/U ratio of 10 or 100) at room temperature for several weeks and did not see any crystallization of U_38_ clusters. A clear green solution was always observed. The formation of the {U_38_} is therefore thermally controlled since it crystallizes in the range of 70–130 °C.

Another factor that could affect the formation of the {U_38_} cluster is the type of alcohol. Several attempts to produce the {U_38_} cluster in different alcohols (ethanol, butanol and cyclohexanol) under the same synthetic conditions (heating at 100 °C for 24 h) did not result in the formation of the {U_38_} cluster and the reaction mixture remained as a clear green solution, which originates from the dissolution of the initial chemicals.

#### SAXS measurements

**Fig. 8:**
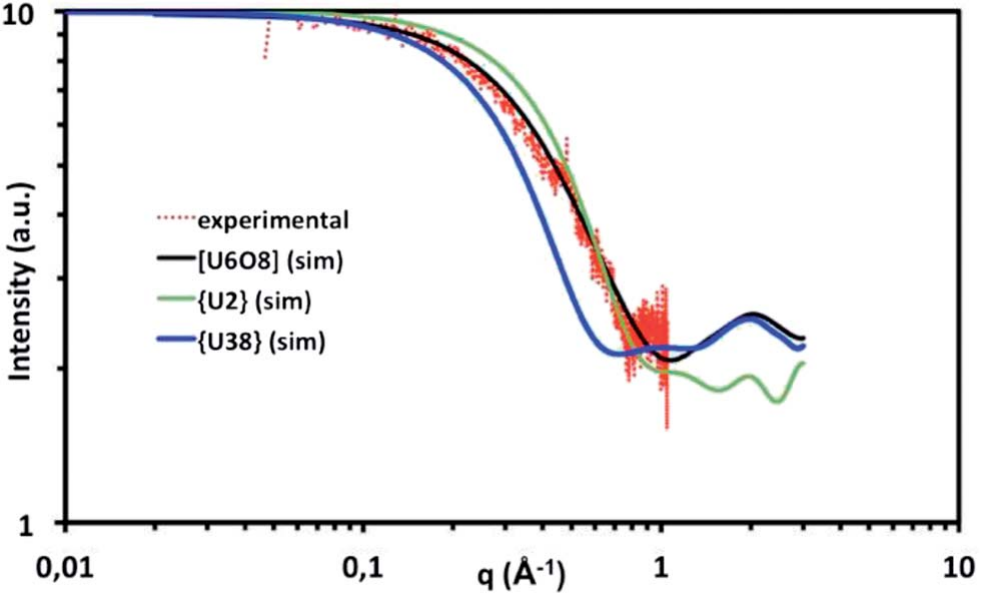
SAXS curve (red plot) for the supernatant obtained after the preparation of the {U_38_} cluster from a mixture of UCl_4_ and benzoic acid in isopropanol, heated for 10 hours at 100 °C, and simulated SAXS curves for [U_2_Cl_4_(bz)_4_(ipa)_4_]·(ipa)_0.5_ (**1**, green solid line), {U_38_} (**2**, blue solid line) and a hexanuclear motif [U_6_O_8_] from Takao *et al.*^23^ (black solid line).

The supernatant obtained from the solvo-thermal treatment was characterized by SAXS for different reaction times from *t* = 0 up to 24 hours, after heating at 100 °C (Hbz/U = 10). The experimental X-ray scattering curve was compared with the model scattering curves simulated for the di-, hexa- and 38-nuclear species of U.^72^ Model scattering curves were simulated by using SolX^73^,^74^ software based on the atomic arrangements of **1** and **2** determined by single-crystal X-ray diffraction. The evolution of the scattering curves (Fig. S10a†) shows the occurrence of low intensity signals for the starting (*t* = 0 hours) and longer reaction times (*t* > 16 hours), indicating the absence of condensed species in the solution. However, from *t* ≈ 4 hours, a signal is clearly observed. The time variation of the scattering intensity follows a bell curve, with the maximum value at around *t* ≈ 8 hours (Fig. S10b†), when the crystallization of the {U_38_} cluster occurs. Fig. 8 depicts the comparison of the experimental scattering curve for the sample supernatant (*t* = 10 hours) with the model scattering curves simulated for the dinuclear species (based on [U_2_Cl_4_(bz)_4_(ipa)_4_]·(ipa)_0.5_**1**) and the poly-oxo {U_38_} cluster (**2**). The scattering curve of the supernatant differs significantly from those simulated for the di- and 38-nuclear species that could potentially occur in the supernatant before the crystal growth process, suggesting that the dominant presence of such oligomer/cluster species with a high concentration is unlikely in the reaction medium and that these entities could be present only in the crystalline state. In order to interpret the SAXS curve obtained for the supernatant, another polymer species of the hexanuclear [An_6_O_8_] complex was considered based on the crystal structure of [U_6_{μ_3_-O(H)}_8_(HCOO)_12_(H_2_O)_6_] reported in ref. 23. This type of polymer complex is well documented for the An(iv) series.^8^ The SAXS curve simulated for the hexameric [An_6_O_8_] unit fits very well with the curve obtained for the supernatant, suggesting that the soluble species in the isopropanol supernatant could exist as a hexanuclear [An_6_O_8_] complex or be of a similar size, which could be stabilized by the coordination of benzoate ligands in the present case. Moreover, the UV-Vis spectral signature (Fig. S5†) of the supernatant is also close to that observed in solution containing the hexamer-bearing [An_6_O_8_] unit, reported in ref. 23. This also suggests that the initial condensation process involves primarily the formation of smaller intermediate species, such as hexamers, and the {U_38_} clusters are not visible in the solution. The formation of molecular bricks with higher nuclearity (*i.e.* {U_38_}) is likely to occur in the nucleation-growth step and the U_38_ occurrence would therefore appear in the solid-state only. It is interesting to notice that the existence of a hexanuclear entity stabilized by the glycine ligand ([U_6_O_4_(OH)_4_(H_2_O)_6_(HGly)_12_]) was also observed in aqueous solution before the precipitation of nanoparticles of UO_2_.^30^ In our case, the presence of the likely hexameric species in solution gave rise to the crystallization to an intermediary state of uranium oxide condensation in isopropanol, with the isolation of the 38-nuclear unit, instead of generating the infinite uranium dioxide network.

## Conclusion

Two new synthesis routes using UCl_4_ and benzoic acid in isopropanol at different temperatures led to the formation of two types of uranium coordination complexes. The synthesis at room temperature results in the formation a dinuclear species ([U_2_Cl_4_(bz)_4_(ipa)_4_]·(ipa)_0.5_, **1**), in which the tetravalent uranium cation (U^4+^) is linked to the adjacent one through four carboxylate bridges of the benzoate molecules. The coordination sphere around each uranium center involves two terminal chloro- and two isopropanol groups, and no direct U–O–U bonding is observed in this dinuclear unit. The other synthesis at a higher temperature produces a second uranium entity (**2**), revealing a poly-oxo cluster bearing 38 uranium centers ({U_38_}). This {U_38_} motif is composed of an internal UO_2_ fluorite-like fragment, to which six faces of the tetranuclear uranium sub-units with chloro or isopropanol groups are attached to its peripheral surface. The structure of the newly obtained {U_38_} cluster is comparable to those of the precedent poly-oxo {U_38_} cluster obtained in THF^6^ and the {Pu_38_} cluster^4^,^5^ with the same nuclearity. Except the difference in elements (U or Pu), the main distinction in this {An_38_} series is the nature of the molecules that are attached on the surface of the clusters. The surface of the cluster exhibits a certain ability to incorporate different coordinating ligands, which can be either neutral (isopropanol, tetrahydrofuran, and water) or negatively charged species (chlorides and carboxylates) with different ratios. Although the formation mechanism of {Pu_38_}^4^,^5^ clusters is reported to be complex and delicate, we successfully synthesized a parent motif of the {U_38_} cluster by using a solvothermal treatment at temperature ranging from 70 to 130 °C. The synthesis strategy employed in this study involved a slow and controlled release of water molecules *via* the *in situ* esterification reaction between a carboxylic acid and an alcohol, eventually inducing the oxolation reaction to generate such a cluster with an extremely high nuclearity based on multiple oxo-bridges between metal centers. Such a synthetic protocol has been successfully employed for the first time with tetravalent actinides for the generation of giant clusters at the nanometer scale (≈1.6 nm). The critical role of the slow release of water in the formation of the {U_38_} cluster was investigated as functions of temperature and carboxylic acid content. It was shown that the direct addition of water in the reaction medium did lead to the formation of a large amount of UO_2_·*x*H_2_O precipitate, together with a few {U_38_} crystallites, revealing the relevant parameter related to the slow kinetics and the requirement of gradual water supply for the formation of the {U_38_} cluster. The SAXS analysis of the supernatant obtained after the production of the {U_38_} cluster showed no clear evidence of the formation of {U_38_} species in solution. But it rather showed the formation of smaller transient entities with a nuclearity close to six, which appear during the early stage of the crystallization process of the compound {U_38_}. This indicates that the {U_38_} motif is stabilized in the solid-state in the crystalline structure, but is also thermally controlled since a temperature in the range 70–130 °C was necessary for its crystal growth process. This study paves the way for the understanding of the formation mechanisms of giant {An_38_} clusters through poly-condensation processes. The generation of such nano-sized cluster species could have a potential impact on the immobilization (migration) of actinides under anoxic conditions relevant to the geosphere.

## Conflicts of interest

There are no conflicts to declare.

## Supplementary Material

Supplementary information

Crystal structure data
