# Supplementary material for: Formation of a new type of uranium(iv) poly-oxo cluster {U38} based on a controlled release of water via esterification reaction
Source: Chem Sci. 2018 May 9;9(22):5021–32. doi: 10.1039/c8sc00752g (PMC5994743; doi:10.1039/c8sc00752g)
Supplement: Supplementary file 1 [file SC-009-C8SC00752G-s001.pdf]

Electronic Supplementary Material (ESI) for Chemical Science.
This journal is © The Royal Society of Chemistry 2018

Formation of a new type of uranium(IV) poly-oxo cluster $\{U_{38}\}$ based on a controlled release of water via esterification reaction.

Nicolas P. Martin,[a] Christophe Volkringer,[a,b] Natacha Henry,[a] Xavier Trivelli,[c] Grégory Stoclet,[d] Atsushi Ikeda-Ohno,[e] and Thierry Loiseau[a,*]

[a]*Unité de Catalyse et Chimie du Solide (UCCS) – UMR CNRS 8181, Université de Lille, ENSCL, Bat C7, BP 90108, 59000 Lille, France*

[b]*Institut Universitaire de France (IUF), 1 rue Descartes, 756231 Paris cedex 05, France*

[c]*Université de Lille, CNRS, UMR 8576, UGSF, Unité de Glycobiologie Structurale et Fonctionnelle, F-59000, France*

[d]*Unité Matériaux Et Transformations (UMET) – UMR CNRS 8207, Université de Lille Nord de France, USTL-ENSCL, Bat C7, BP 90108, 59652 Villeneuve d'Ascq, France*

[e]*Helmholtz-Zentrum Dresden-Rossendorf, Institute of Resource Ecology, Bautzner Landstrasse 400, 01328 Dresden, Germany*

[*] To whom correspondence should be addressed. E-mail: thierry.loiseau@ensc-lille.fr. Phone: (33) 3 20 434 122, Fax: (33) 3 20 43 48 95.

SUPPLEMENTARY MATERIALS

Sections:

1. Optical microscopy of compounds 1 and 2

2. Esterification reaction scheme

3. Checkcif comments

4. Disorder of isopropanol molecules in complex 1, $[U_2Cl_4(bz)_4(ipa)_4]\cdot(ipa)_{0.5}$

5. Bond valence calculations

6. Thermogravimetric analysis

7. UV-Vis spectroscopy

8. Avrami-Erofe'ev and Sharp-Hancock kinetic treatments of crystallization of $\{U_{38}\}$

9. ^{1}H NMR spectra as a function of time

10. Evolution of the reaction yield as a function of benzoic acid/U ratio

11. SEM photographs of powdered by-products obtained during the synthesis of $\{U_{38}\}$

12. Powder X-ray diffraction patterns of the powdered by-products obtained during the synthesis of $\{U_{38}\}$

13. SAXS curve of the supernatant solution at different reaction times for the synthesis of $\{U_{38}\}$

1. Optical microscopy of compounds 1 and 2

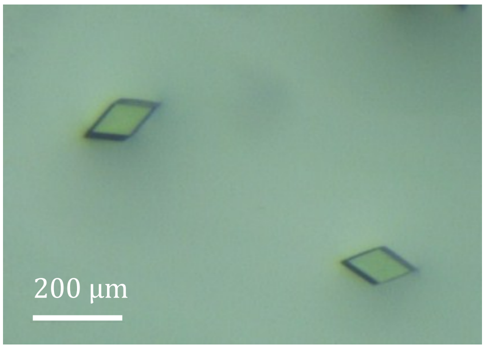

Figure S1a: Optical photographs of crystals of compound **1** ($[U_2Cl_4(bz)_4(ipa)_4]\cdot(ipa)_{0.5}$) obtained at room temperature.

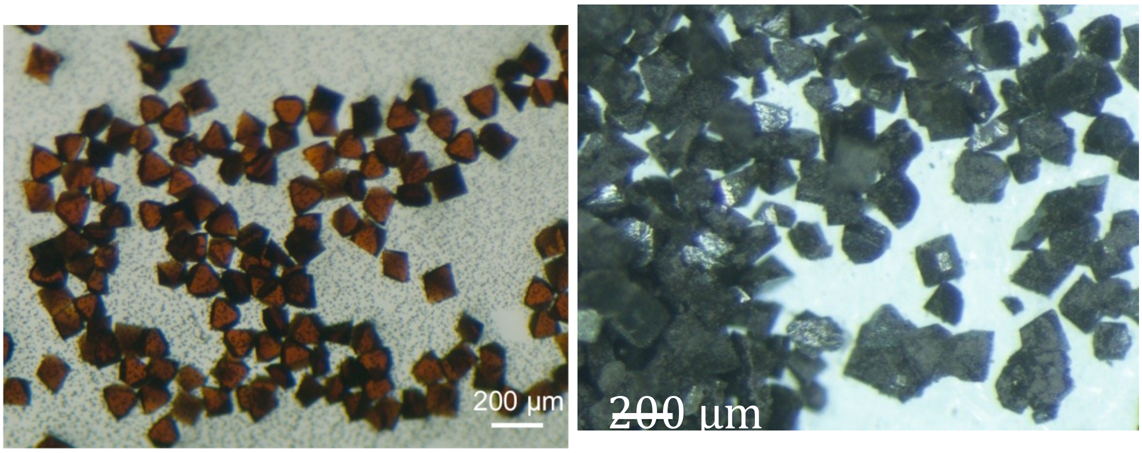

Figure S1b: Optical photographs of crystals of **2** ($[U_{38}O_{56}Cl_{40}(H_2O)_2(ipa)_{20}]\cdot(ipa)_x$). left : $\{U_{38}\}$ in the mother liquor; right : $\{U_{38}\}$ crystals exposed to air.

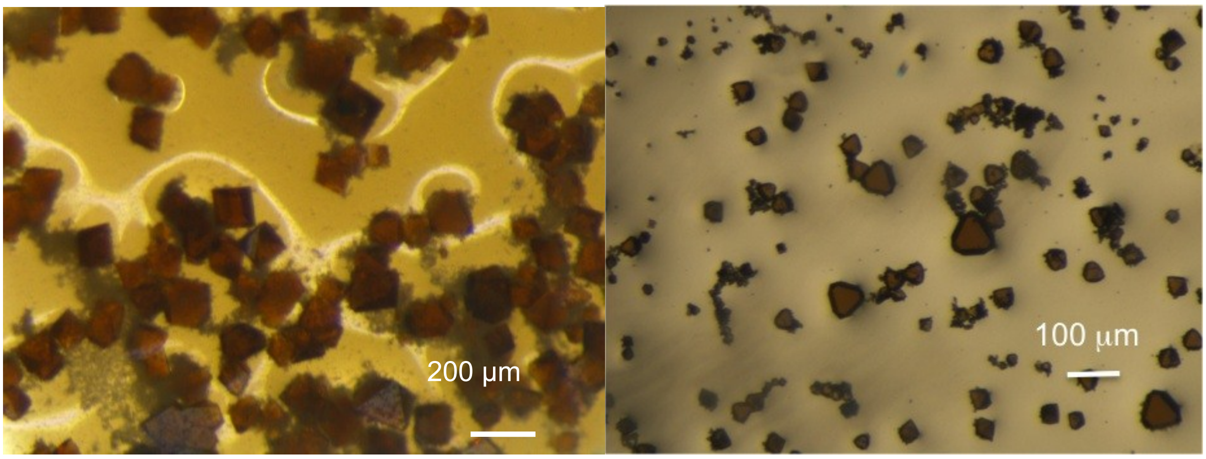

Figure S1c: Optical photographs of crystals of **2** ($[U_{38}O_{56}Cl_{40}(H_2O)_2(ipa)_{20}]\cdot(ipa)_x$) obtained with methacrylic acid (left) and formic acid (right), instead of using benzoic acid.

2. Esterification reaction scheme

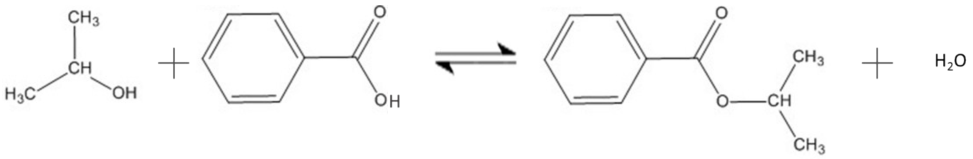

Figure S2: Classical scheme of the reaction of benzoic acid with isopropanol resulting in the formation of an ester and the release of one water molecule.

3. Checkcif comments

Comments on the Alert A in the checkcif file for the compounds **1** and **2**.

For **1**, the alert A correspond to a problem for converging one H atom of the disordered isopropanol molecule attached to the ligand ("B" backbone), due to its proximity of other neighboring H atoms induced by the disordering situation.

For 2, The six alerts A in the checkcif file concern residual density located on the heavy uranium atoms (about 3.2-2.2 e.Å^{-3}). This abnormally values would be due to the fact that the crystal has been enveloped with a too large amount of grease in order to prevent any decomposition under air atmosphere. It induces some problems with adsorption and diffusion processes, which interfere on the electron density map.

4. Disorder of isopropanol molecules in complex 1, $[U_2Cl_4(bz)_4(ipa)_4]\cdot(ipa)_{0.5}$

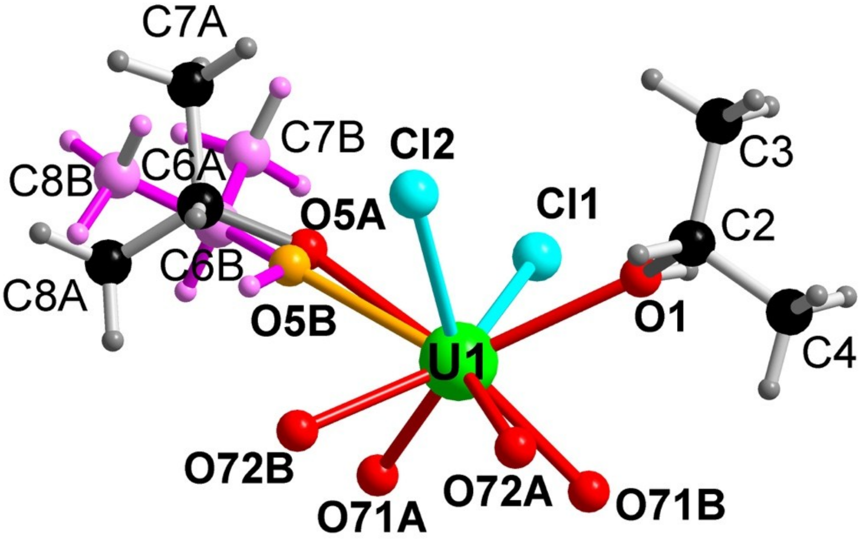

Figure S3a: Detailed view of the disorder of the attached isopropanol ligands to the uranium center U1 in complex **1**. One of the two isopropanol molecules is found disordered on two close positions with the ratio 75/25 for the "A'(grey bonds) and "B" (purple bonds) backbones, respectively.

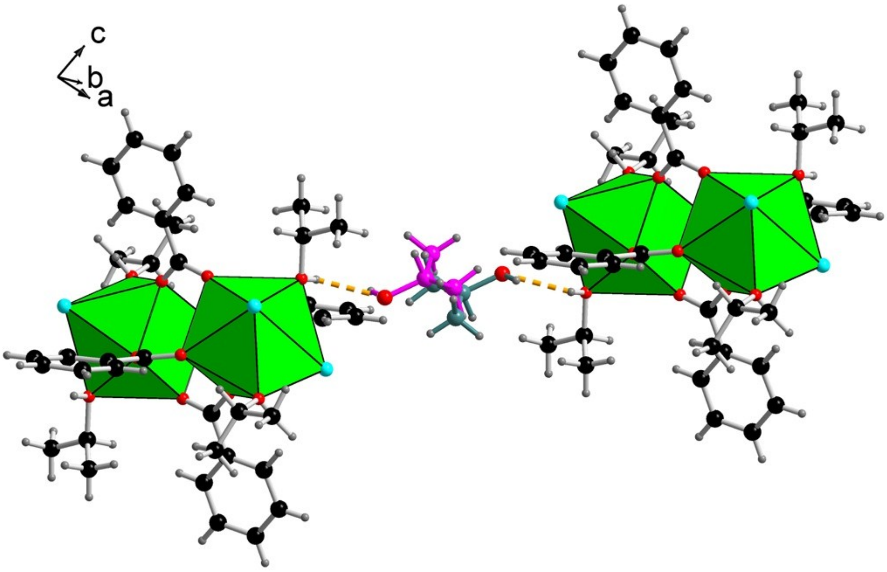

Figure S3b: Representation of the disordered isopropanol molecules intercalated between two dinuclear entities $U_2Cl_4(bz)_4(ipa)_4]$, in complex **1**. This isopropanol species has been refined with an occupancy factor of 0.25, and is located on two close positions. Hydrogen bonds interactions (dotted orange line) are observed between the OH groups of the attached isopropanol, and OH groups of the isopropanol.

5. Bond valence calculations

Table S1. Bond valence sum (= exp[(R_o - R)/B]) for ([$U_{38}O_{56}Cl_{40}(H_2O)_2(ipa)_{20}$]·$(ipa)_x$

	R_o	B	References
**U-O**	2.112	0.37	[1]
**U-Cl**	2.48	0.37	[1]

**Atom**	**Bond valence**	**Atom**	**Bond valence**	**Atom**	**Bond valence**
**U1**	**4.059**	**Cl2**	**0.831**	**O3**	**2.015**
**U2**	**4.08**	**Cl3**	**0.548**	**O4**	**2.085**
**U3**	**4.019**	**Cl4**	**0.735**	**O5**	**2.076**
**U4**	**3.905**	**Cl5**	**0.532**	**O6**	**2.004**
**U5**	**3.969**	**Cl6**	**0.798**	**O1aa**	**2.065**
**U6**	**4.084**	**Cl7**	**0.781**		
**U7**	**4.168**	**O1**	**2.039**		
**Cl1**	**0.765**	**O2**	**2.095**		

The bond valence sum is expected to be 2.0 for an oxo group, 1.2 for a hydroxo group and 0.4 for an aquo group.

[1] Brese and O'Keeffe, (1991), Acta Cryst. B47, 192-197

6. Thermogravimetric analysis

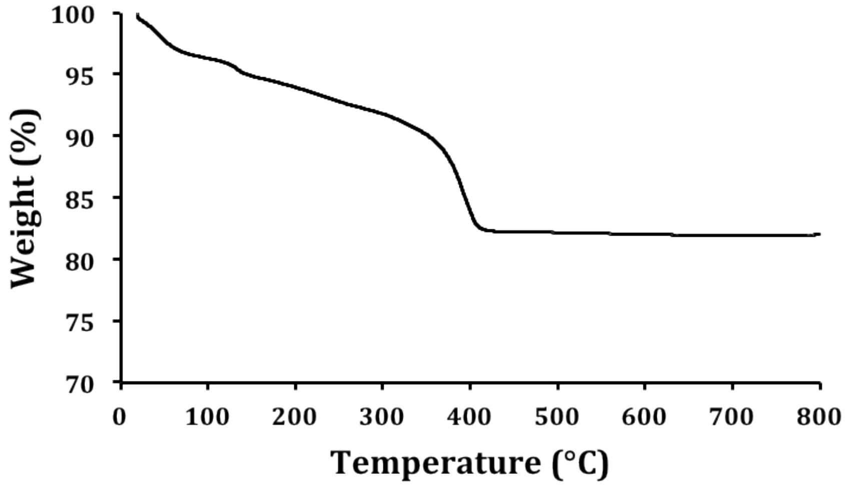

Figure S4: Thermogravimetric curve of compound **2**, $\{U_{38}\}$, under air atmosphere (heating rate: 5°/min).

7. UV-Vis spectroscopy

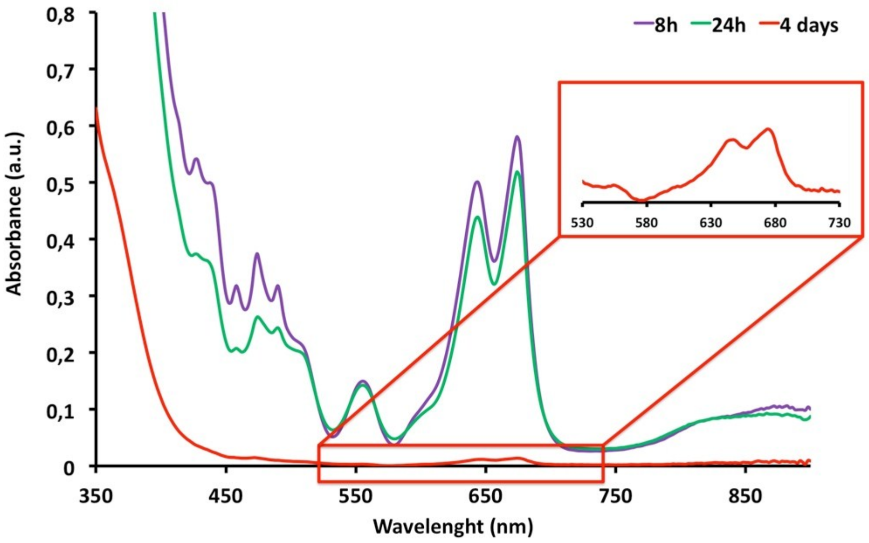

Figure S5: UV-vis spectra of the liquid supernatant resulting from the formation of $\{U_{38}\}$ after the solvothermal treatment at 100°C for 8h (violet curve), 24h (green curve) and 96h (red curve).

The UV-Visible absorption spectra were measured for the supernatants obtained after the solvothermal treatment at 100 °C with different reaction times (8, 24 and 96 h) (Figure S4). The spectra indicate strong absorption bands in the range between 580 and 720 nm, which are typically assigned to the electronic transitions of $^3H_4 \rightarrow ^3P_0$, $^3H_4 \rightarrow ^1G_4$ and $^3H_4 \rightarrow ^1D_2$ of U(IV). Indeed, two intense bands are appeared at 644 and 674 nm together with a shoulder at 602 nm. Another significant absorption is also clearly visible at 560 nm, which is assigned to the electronic transitions of $^3H_4 \rightarrow ^3P_1$. The intensities of these bands slightly decreased then the reaction time increased from 8 to 24 h, which is related to the reaction yields of 3.3 and 36.7%, respectively. The intensities further decreased when the reaction time was 96 h, corresponding to the yield of 80.9%. This reflects the consumption of soluble uranium species for the crystallization of the $\{U_{38}\}$ cluster.

8. Avrami-Erofe'ev and Sharp-Hancock kinetic treatments of crystallization of {U_{38}}

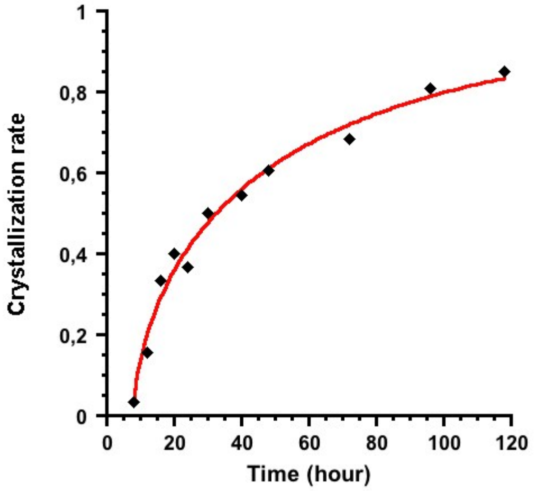

Figure S6a: Kinetic curve of the crystallization rate of the {U_{38}} polyoxo cluster (heating at 100°C), between 8 and 120 hours. Black lozenges: experimental data; red curve: calculated fit using the Avrami Arofe'ev equation.

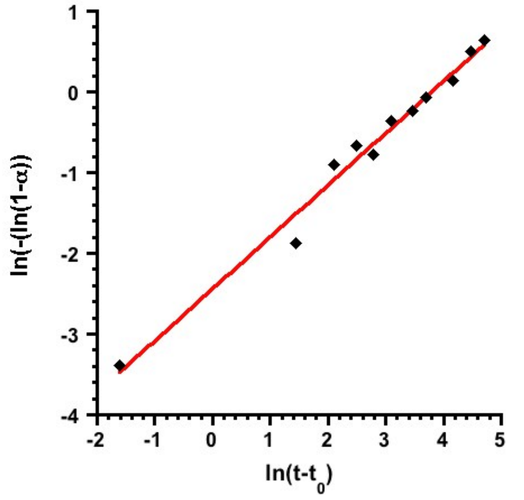

Figure S6b: Sharp-Hancock plot from linear regression of the Avrami-Erofe'ev equation ($\ln[-\ln(1-\alpha)] = n\ln(k) + n\ln(t\text{-}t_0)$), for the crystallization of {U_{38}} from the solvothermal reaction at 100°C.

9. ^{1}H NMR spectra as a function of time

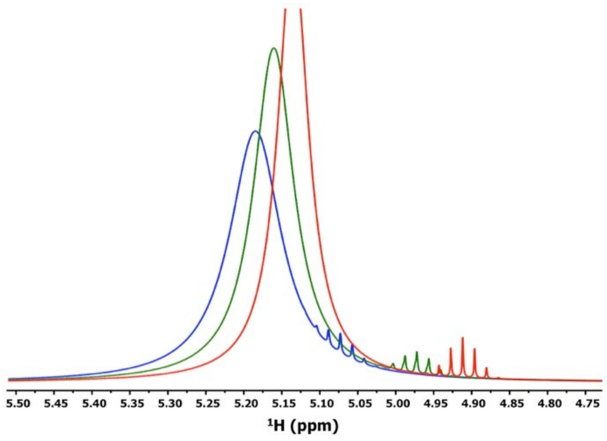

Figure S7: ^{1}H NMR spectra of the supernatants obtained after the solvothermal reactions to produce the $\{U_{38}\}$ cluster at 100°C for 8 (blue), 16 (green) and 24 hours (red). The data were recorded at 400 MHz and 285K. The range corresponds to the free –OH group of isopropanol (broad signal) and –CH groups of the ester molecule (weak multiplet signals).

10. Evolution of the reaction yield as a function of benzoic acid/U ratio

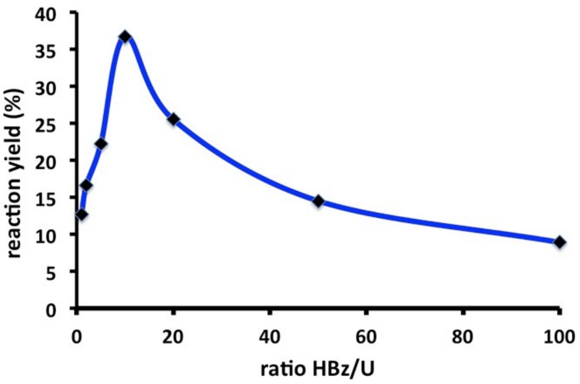

Figure S8: Change of the reaction yield for the crystallization of $\{U_{38}\}$ as a function of benzoic acid/U molar ratio.

11. SEM photographs of powdered by-products obtained during the synthesis of $\{U_{38}\}$

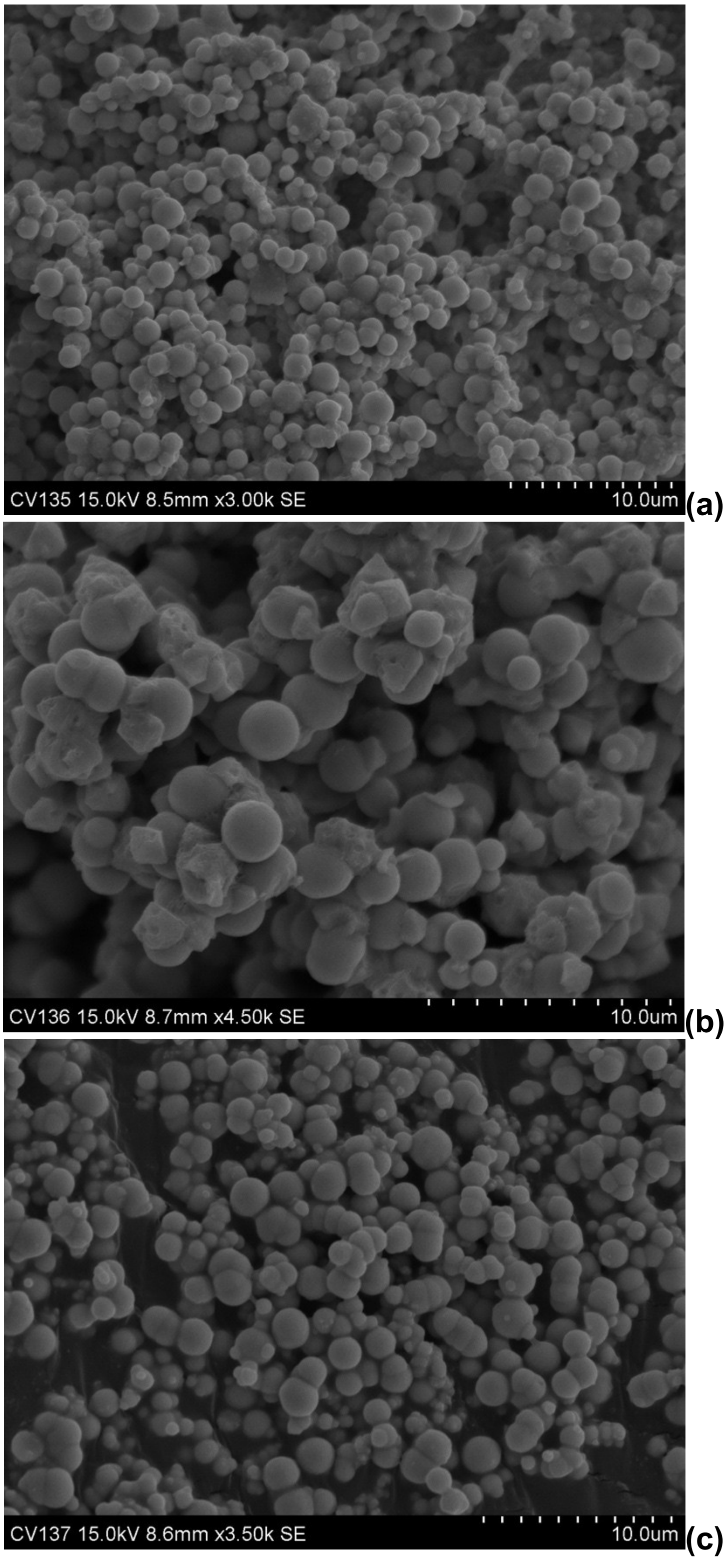

Figure S9a: SEM photographs of the powdered precipitate from the reaction of UCl_4 with water in isopropanol solvent, after heating at 100°C. (a) addition of 5 μL H_2O; (b) addition of 25 μL H_2O – small octahedrally shaped crystals related to the $\{U_{38}\}$ cluster are observed together with spherical particles; (c) addition of 50 μL H_2O.

12. Powder X-ray diffraction patterns of the powdered by-products obtained during the synthesis of {U_{38}}

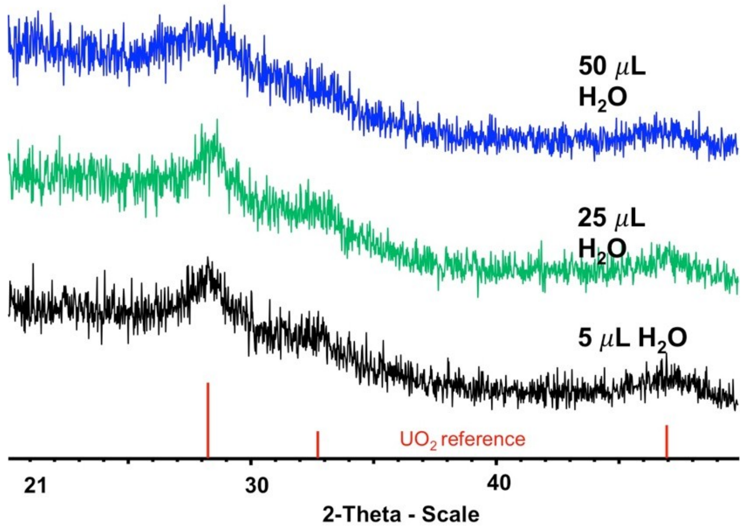

Figure S9b: Powdered X-ray diffraction patterns (copper radiation) of the resulting precipitate from the reaction of UCl_4 with different amounts of water in isopropanol solvent, after heating at 100°C. The reference of the dense uranium dioxide UO_2 with the fluorite-structure type (PDF No 03-065-0285) is indicated in red, for comparison.

13. SAXS curve of the supernatant solution at different reaction times for the synthesis of $\{U_{38}\}$

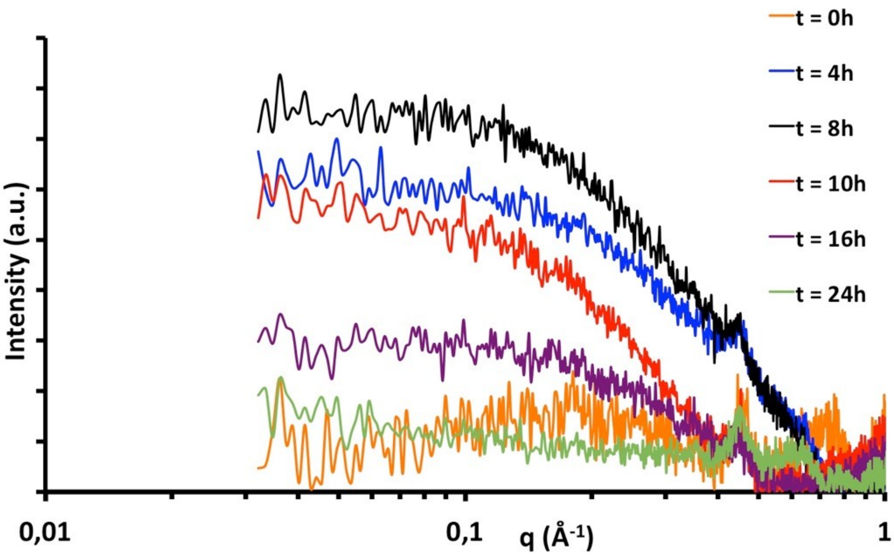

Figure S10a: Evolution of the room temperature SAXS curves of the supernatant as a function of time (t = 0h, 4h, 8h, 10h, 16h, 24h). The liquid phase analyzed by SAXS corresponds to the resulting solution from the mixture of UCl_4 and benzoic acid in isopropanol after heating at 100°C.

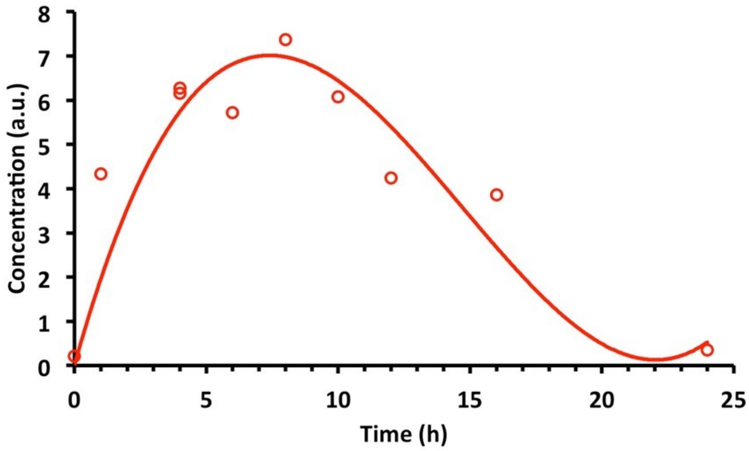

Figure S10b: Time-dependent evolution of the concentration of condensed species in the supernatant derived from the SAXS curves collected after heating at 100 °C. The data were collected at room temperature.
